# Complications and satisfaction in transwomen receiving breast augmentation: short- and long-term outcomes

**DOI:** 10.1007/s00404-022-06603-3

**Published:** 2022-05-21

**Authors:** A. K. Schoffer, A. K. Bittner, J. Hess, R. Kimmig, O. Hoffmann

**Affiliations:** 1 Department of Gynecology and Obstetrics, University Hospital Essen, Hufelandstraße 55, 45147 Essen, Germany; 2 Department of Urology, University Hospital Essen, Essen, Germany

**Keywords:** Transwomen, Breast augmentation, Capsular contracture, Satisfaction, Breast implants

## Abstract

**Background:**

To achieve long-term improvement in health care of transgender women, it is necessary to analyze all aspects of gender-confirming surgery, especially the relation of risks and benefits occurring in these procedures. While there are many studies presenting data on the urologic part of the surgery, there are just few data about complications and satisfaction with breast augmentation.

**Methods:**

This is a retrospective study using parts of the BREAST-Q Augmentation Questionnaire and additional questions for symptoms of capsular contracture and re-operations and analyzing archived patient records of all transwomen which were operated at University Hospital Essen from 2007 to 2020.

**Results:**

99 of these 159 patients (62%) completed the questionnaire after a median time of 4 years after surgery. Breast augmentation led to re-operations due to complications in 5%. The rate of capsular contracture (Baker Grad III–IV) in this population was 3%. Most patients (75%) rated high scores of satisfaction with outcome (more than 70 points) and denied to have restrictions due to their implants in their everyday life. All patients reported an improvement in their quality of life owing to breast augmentation.

**Conclusion:**

Breast augmentation by inserting silicon implants is a safe surgical procedure which takes an important part in reducing gender dysphoria.

## Introduction

Many male-to-female transgender individuals (transwomen) seek surgical feminizing procedures throughout their lives to improve their gender dysphoria and quality of life. An important part of feminization is developing natural shaped, feminine breasts [1] which psychologically plays a central role in the femininity, attractiveness and sexuality of women [2]. However, cross-sex hormone therapy (CSHT), which is often performed initially, usually results in inadequate breast growth that often does not even correspond to cup size AA and does not change after the first six months of therapy [3]. Therefore many transwomen search for surgical breast augmentation [4]. Although gender-affirming surgery (GAS) has become more common in recent years, there have been few studies addressing complications and satisfaction of surgical breast augmentation [4–9]. Especially regarding the fact, that transwomen are often older, have a higher BMI and suffer from pre-existing medical conditions, it is very important to analyze risks and benefits of any surgical procedure [10]. In addition, there are specific risks associated with implant surgery, such as capsular contracture [11] or breast implant-associated lymphoma (BIA-ALCL) [12, 13]. Consideration should also be given to the potentially increased risk of developing breast cancer due to CSHT [14] and the limited validity of imaging due to the insertion of silicone implants [15].

In recent years, patient-reported outcome measures (PROM) have been increasingly used to measure the benefit of a surgical intervention. The BREAST-Q is a PROM that is available in three different modules for augmentations, reconstructions and reductions of the breast [16] and has been validated [17]. However, this questionnaire was created for genetic women, and a PROM developed specifically for the needs of transgender individuals does not exist to date [18, 19] As GAS increases worldwide, in 2018, a working group from Canada, the United States, and the Netherlands began a Phase I study and development of a GENDER-Q questionnaire [20], which is currently in the validation phase and is expected to be available in 2022 [16].

The available literature measures satisfaction with surgical breast augmentation either using the BREAST-Q [9] or with help of specifically designed PROMs [4, 18]. Kanhai et al. and Weigert et al. demonstrated a high level of satisfaction with the outcome of augmentation, but did not record any surgical complications [4, 9]. Balakrishnan et al. conducted a study in which they asked a total of 42 transwomen about their subjective satisfaction in a retrospective study from 2007 to 2017 and then compared this with objectively recorded parameters of cosmetic outcome. They showed a significant correlation of the cosmetic outcome with the surveyed parameters [5], but this must be critically questioned, as patients with psychiatric pre-existing conditions, nicotine or drug use and other comorbidities (diabetes, hypertension, vasculitis) were excluded from the study. Nauta et al. compared 82 trans-women to 188 genetic women in America and were able to show differences in comorbidities and anatomical conditions [10]. No study has yet succeeded in identifying risk factors for the occurrence of specific complications, such as capsular contracture, in the context of surgical breast augmentation for transwomen.

### Aim of this study

The aim of this study was to interview all transwomen who underwent breast augmentation at University Hospital Essen between 2007 and 2020 using parts of the BREAST-Q Augmentation Questionnaire, which was supplemented by questions asking for re-operations and high-grade capsular contracture. In addition, the archived patient records were searched for comorbidities and surgical complications. Another purpose was to investigate the occurrence of higher-grade capsular contracture and try to identify risk factors.

## Methods

First, the study design was approved by the ethics board of University Hospital Essen and prior to participating, all patients provided their informed consent. Subsequently, a questionnaire was designed, which consisted of parts of the postoperative BREAST-Q augmentation questionnaire (Psychosocial Wellbeing, Sexual Wellbeing, Satisfaction with Breasts, Physical Wellbeing: Chest, Satisfaction with implants, Satisfaction with outcome) and was supplemented by questions about complications, re-operations and symptoms for capsular contracture. This questionnaire was sent to all 159 transwomen who had received surgical breast augmentation using silicone implants at University Hospital Essen from 2007 to 2020. Of these 159 patients, 99 agreed to participate in this study (62%). In addition, the archived patient records of these 99 patients were reviewed and age, BMI, pre-existing conditions, and surgical complications were recorded.

Data were collected and analyzed using Microsoft Excel 365 (Microsoft Corporation, version 2102, Redmont WA, USA) and IBM SPSS Statistics (IBM Corporation, 2020. IBM Statistics for Windows, version 27.0. Armonk, NY, USA). BREAST-Q was converted to the corresponding Rasch sum score.

To compare satisfaction scores by age, the Shapiro–Wilk test searching for normal distribution was first performed. Since there was no normal distribution, the Mann–Whitney *U* test was used. The significance level was set at *p* < 0.05.

## Results

**Fig. 1:**
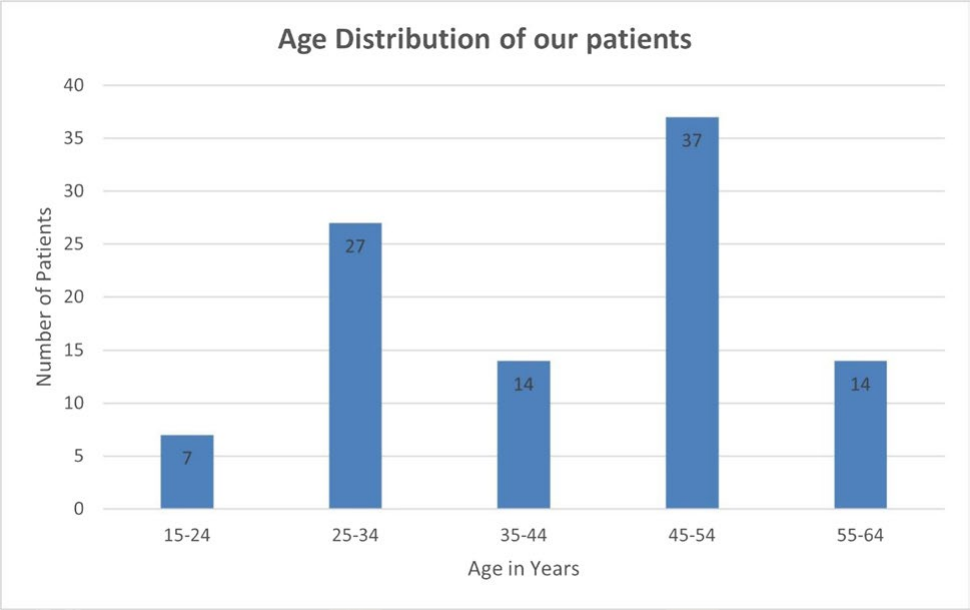
Age distribution of our patients

The median age of the patients at the time of surgery was 45 years (20–64 years, SD 11.63). Using a graphical representation of the age distribution, two peaks can be identified, between 25 and 34 years and between 45 and 54 years (Fig. 1).

At the time of the study, surgery had been performed a median of 4 years ago (0–13 years, SD 3.22). The median BMI of the patients was 24.9 kg/m^2^ (18.0–42.4 kg/m^2^, SD 4.74). 68.7% of patients reported not smoking, 30.3% were smokers, and 1% were unknown. 3% of patients suffered from diabetes. 56.6% of the patients suffered from one or more pre-existing condition, the most frequently mentioned being hypertension (20.2%), asthma (9.1%), hypothyroidism (8.1%), depression (7.1%), previous myocardial infarction (5.1%), sleep apnea (3%), COPD (3%), and previous thrombosis (3%). All other mentioned pre-existing conditions occurred in less than 3% of the patients. 91.9% of the patients stated that they regularly took one or more medications, 7.1% stated that they did not take any medications—although it is no longer possible to evaluate whether this is due to an inaccurate medical history and the patients did not consider their hormone therapy as medication or really did not take any medication. 86.9% of the patients reported receiving estrogen for CSHT.

At University Hospital Essen, textured implants are used exclusively: 67.7% of patients received implants from Mentor, 27.3% from Allergan, 3% from Rofil Medical, 1% from Poly Implant Prothése (PIP) and in 1% the manufacturer was unknown.

Table 1 shows the average implant sizes used. 74.8% of the implants were implanted prepectoral and 25.2% below the muscle.

**Table 1:** Average implant sizes

Manufacturer	Mean	Min; max	SD
Mentor/PIP (cc)	365	195; 650	104.4
Allergan (g)	363	210; 695	101.5
Rofil (g)	323	230; 470	128.6

A total of 10 re-operations were performed in University Hospital Essen, the largest proportion (70%) of which involved the replacement of implants from the companies PIP and Rofil Medical after it became public, that these companies used inferior industrial silicone for their implants [21]. 1% required relief of a hematoma, 1% developed a late hematoma after several months, and 1% developed bilateral Baker grade III–IV capsular contracture, so that both implants had to be removed without replacement. 4% of the patients stated that they had undergone further surgery in other hospitals. 1% had a seroma to be surgically relieved, 1% had a dislocated implant, which was replaced, and 1% had implants replaced for cosmetic reasons. This results in a reoperation rate due to surgical complications of 5% for the presented collective.

When asked about symptoms of grade III–IV capsular contracture, two patients responded that they had symptoms, and one patient stated that her implants had already been removed due to capsular contracture. This results in a rate of 3% capsular contracture Baker grade III–IV.

**Fig. 2:**
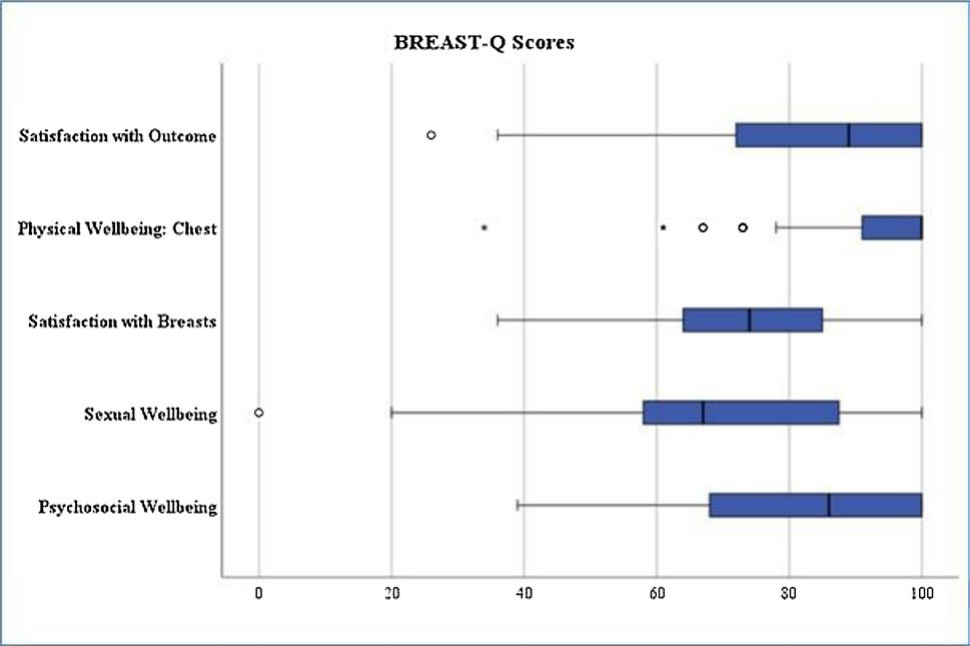
BREAST-Q scores

In addition, the patients answered selected questions of the postoperative BREAST-Q questionnaire. Figure 2 shows graphically the points remitted by the patients, Table 2 lists all calculated values.

**Table 2:** Calculated values from BREAST-Q

	*n*	Mean	Median	SD	Min	max	1. Quartil	3. Quartil
Psychosocial wellbeing	98	81.2	86	17,587	39	100	68	100
Sexual well being	87	70.33	67	22,333	0	100	58	91
Satisfaction with breasts	99	73.72	74	15,402	36	100	64	85
Physical wellbeing: chest	99	92.49	100	10,756	34	100	91	100
Satisfaction with implants	98	7.66	8	1025	2	100	8	8
Satisfaction with outcome	99	83.51	89	18,442	26	8	69	100

There is no conversion for the questions on satisfaction with the implants; patients can give 2–8 points here. 86.7% of the patients awarded a full 8 points.

As shown in Fig. 1, two age peaks can be identified in the examined collective which therefore can be divided into two groups of approximately equal size: Patients who were between 20 and 44 years old (*n* = 48) and patients who were 45–64 years old (*n* = 50) at the time of surgery. The Mann–Whitney *U* test was used to determine whether the older patients were more satisfied with the outcome of surgery. The results show, that the group of patients over 45 years old gave significantly higher scores for psychosocial well-being (*p* = 0.032) and physical well-being (*p* = 0.010). Satisfaction with outcome also showed a tendency toward better satisfaction in the group of older patients with *p* = 0.054.

## Discussion

The age distribution in this study is similar to that of comparable studies [22, 23], and the distribution curve with two peaks is also found in other studies on GAS [23, 24]. While Jackowich [24] and Zavlin [23 attribute this mainly to economic factors, the patient records of our population also showed another possible reason: During the psychological evaluation, many patients reported a desire to have children and the fear of not being able to realize this desire in a life as transwoman. This also seems to be a possible explanation, why a large number of patients decided to live out their true gender identity openly later in life.

The overall rate of re-operations is 14%. The largest proportion of this, however, is based on the replacement of PIP/Rofil implants, which accounts for a total of 8% of re-operations and can certainly not be seen as a general complication of implant surgery, since it is a matter of poor product quality of only a few manufacturers. 1% voluntarily underwent another operation for cosmetic reasons to have their breasts enlarged even further. All patients receive estrogen therapy for at least one year to achieve pre-expansion of the skin and maximum self-growth of the breast prior to surgery [25], yet the implants can only be large enough to still be covered by skin, adipose tissue, and muscle if necessary. Thus, initially, it is not always possible to meet patients' desires for a very large breast. Fakin et al. [8] also reported 9.4% patients (13 of 138) who underwent a second surgery due to the desire for a larger breast. 1% of our patients experienced postoperative bleeding, 1% experienced late hematoma, 1% experienced unilateral dislocation, 1% experienced seroma after hospital discharge, and 1% developed capsular contracture, which also required reoperation. This results in a reoperation rate of 5% due to complications, which is slightly lower than the 8% non-elective surgery rate reported by Fakin et al. [8]. Comparing these data to those collected by Cuccolo et al. [6], who conducted the largest retrospective data analysis comparing breast surgery in transwomen and genetic women, reveals a higher rate of complications. Cuccolo et al. [6] report a rate of 1.4% re-operations in transwomen (four cases in a total of 280 patients), accounted for by hematoma (1.1%) and abscess (0.4%). However, when looking at these data, it is important to note, that this is a large database analysis in the U.S., and while it draws on a large number of cases, it only captures complications within the first 30 days after surgery and does not capture any long-term complications.

In comparison, the data collected by Miller et al. [18] show a higher complication rate of 17.6% (6 of 34 cases) in which reoperation was necessary in 5.9% (2 of 34 cases). However, the significance of this should also be questioned, as only 34 cases were retrospectively examined in this study.

The largest data analysis for genetic women is provided by Coroneos et al. [26], who were required to provide retrospective data collection for FDA approval of Mentor and Allergan breast implants and studied a total of 99,993 patients from 2007–2017, 56% of whom had received silicone implants as part of a purely cosmetic procedure [26]. Here, an overall reoperation rate of 11.7% at 7 years was reported, which is higher than the 5% in transwomen collected in this study. Coroneos et al. [26] also report a Baker III-IV capsular contracture rate of 7.2% at 7 years for cosmetic augmentations in genetic women. Since these data refer exclusively to implants from the Mentor and Allergan companies and implants from these two companies were also used to a large extent in our cohort, and furthermore the comparison period of 10 and 13 years is similar, these data were considered to be the most meaningful and were used as basis. It should be noted, that Coroneos et al. [26] do not break down the described complication rates according to smooth or textured surface, whereas only textured implants are used at our clinic.

A significantly higher rate of complications is described by DeBlok et al. [7] in their study conducted on a Dutch collective. There, 33% (102 of 308 patients) reported suffering from health complaints associated with their implants [7]. Unfortunately, this statement is difficult to verify as the authors of the study do not provide a more detailed breakdown of what exactly constitutes health complaints and, although the study was conducted on a large collective (3074 transwomen) and over a long period of time (1972–2018), the response rate is only 25.14% with 773 completed questionnaires. Furthermore, it is likely that even worse implants were used in the 1970s and 1980s than is the case today, possibly leading to a falsely high complication rate. To derive a correlation between the complications that occurred (postoperative bleeding, late hematoma, seroma as well as unilateral dislocation) and risk factors, such as smoking, diabetes, pre-existing diseases or long-term medication, too few cases occurred within the scope of our study, to be able to derive a conclusion here.

3% of patients reported receiving a diagnosis of capsular contracture, of which 1% had already had their implants removed. Taking this 3% as the rate of symptomatic capsular contracture on average 5 years after surgery and comparing it, using the binomial test, with the 7.2% symptomatic capsular contracture at 7 years described by Coroneos et al. [26] yields a value of *p* = 0.071, showing a tendency toward a lower rate of capsular contracture in transwomen. Similar findings are also provided by Fakin et al. [8], who reported a capsular contracture rate of 2.9%. To further support this theory, more studies on this topic should be conducted in future.

Estrogen exposure seems to be the first possible reason for a lower rate of capsular contracture. This assumption is supported by data presented by Dancey et al. [27], who showed that in a cohort of 1400 genetic women who underwent cosmetic augmentation, those who became pregnant had an increased risk of developing capsular contracture (43.6% vs 23.9% capsular contracture, *p* = 0.008) [27]. Since there is also greater exposure to estrogen during pregnancy, it is reasonable to suspect a central role in the etio-pathology of capsular contracture. This is also consistent with data collected by Persichetti et al. [28], according to which downregulation of ER α leads to decreased contractility of myofibroblasts. They also showed a negative correlation between ER β and the thickness of the capsule studied, suggesting that ER β has a more antiproliferative role [28]. Moreover, this study also showed a time-dependent correlation, which was also supported by Joseph et al. [29] in a study performed in rats. Accordingly, the concentration of pro-proliferative factors is significantly higher 30 days after implantation, than 90 or 180 days after implantation [29]. A perioperative pause of CSHT in transwomen may decrease the development of capsular contracture. Maybe the administration (oral vs transdermal) or the active ingredient composition of the preparation also affects the development of capsular contracture in transwomen.

Prospective randomized studies should be performed on these influences in future, recording the exact estrogen preparation, the duration of intake and the perioperative pause, to investigate these influences in more detail and thus possibly provide further insights into the development and the prevention of capsular contracture.

In addition, studies should be performed to break down the rate of capsular contracture in transwomen after insertion of smooth or textured implants. Due to the rare occurrence of BIA-ALCL after insertion of textured implants [30], some authors generally do recommend not to use this implants any more [31]. To date, only four cases of BIA-ACLC have been reported in transwomen worldwide [12], so the incidence here is certainly more in the per thousand range, whereas the risk of high-grade capsular contracture with possible definitive implant removal and breast firming surgery is at least in the single-digit percentage range and is thus significantly greater. However, since a surgical breast augmentation for transwomen is not a purely cosmetic procedure, all risks should be carefully weighed.

This becomes even clearer with a look at the level of satisfaction. The median psychosocial well-being of the patients studied here was 86 of 100 points. This observation is consistent with the results of Weigert et al. [9], who conducted the only study using the BREAST-Q in transwoman and who found the patients' psychosocial well-being to be median 85 points 4 months after surgery.

Another important aspect of breast surgery is sexual well-being, although many patients stated that this was also very significantly dependent on the genital surgical outcome, which leads to a certain outcome bias. Although a median score of 67 was given, 25% of patients declared a satisfaction score of 91 or more.

The median satisfaction with the breast was 74 points, which means that the patients in this study were significantly more satisfied than those surveyed by Weigert et al. [9]. When looking at the questions individually, many patients, when asked how satisfied they were with the way their bras fit, expressed a certain dissatisfaction and stated, that it was very difficult to find bras at all, due to the larger breast circumference. While this is not a medical problem, it is certainly an industrial market gap.

Furthermore, 30% of the patients were dissatisfied with the fact that the breasts were not as close together without a bra. This is a peculiarity of the anatomically male developed upper body, which has a narrower, more oval and laterally seated nipple–areola complex (NAC) and more developed pectoralis muscles than the female. In addition, the sternum is much wider and the distance between the nipple and inframammary fold is much shorter [1]. Unfortunately, the insertion of silicone implants cannot remedy this situation.

The high scores in relation to physical well-being indicate, that the patients have few impairments overall, such as limitations in sports, lifting heavy objects or sleeping, due to the implants or complications of surgery. This results clearly differ from data collected by Weigert et al. [9], in which their patients gave a median score of only 76 points.

In terms of satisfaction with the implants, patients gave a median of full 8 points, which underlines the satisfaction with implant surgery itself.

Satisfaction with outcome was very high, with a median score of 89 points, and 25% of patients awarded the maximum of 100 points. Particularly noteworthy is the question about the improvement in quality of life, to which 89% of the patients agreed completely. A further 11% stated that they agreed to some extent, and no patient stated that she did not agree. Even though this question alone certainly does not do justice to a multi-layered construct as quality of life, but together with the other values collected, it very clearly shows the high value that breast augmentation has for transwomen.

Furthermore, it was shown here that older transwomen seem to be even more satisfied with the results. The reasons for this may be, that older patients have not spent a larger proportion of their lifetime in the desired sex and therefore have an overly positive view of the effect of breast augmentation. It is also possible, that with increasing physiological aging of the body, the demand for perfection becomes lower and older patients are therefore less critical with the cosmetic result.

95% of the patients stated, that they did not regret the operation, only 3% would not undergo the operation again. This is possibly due to complications suffered and associated discomfort, as Cash et al. [32] were able to survey in a study of non-transgender women after surgical breast reconstruction and as also mentioned by the transwomen interviewed by DeBlok et al. [32, 33].

### Limitations

The response rate of 62% is slightly above the existing literature. Weigert et al. [9] had recruited only 35 patients for their study from the outset, of whom only 60% answered the questionnaire sent to them after just one year. Kanhai et al. [4] were able to include 58.2% in their study and Miller et al. [18] were only able to achieve a response rate of 35.3% for their questionnaire.

Two patients stated that they did not wish to participate in our study, and in a total of 57 patients the address or telephone number was no longer correct, which is not surprising given a period of 13 years. It seems reasonable to assume that some patients moved after successful GAS, to live a completely new life in a different place in the desired gender role without being reminded of their old life. It also seems conceivable that some patients did not complete the questionnaire in order be not reminded of their old life and the transition steps [4] or possibly because they regretted the surgery [34].

One patient of our collective committed suicide, which is also a possible reason for the lack of responses from other patients, because, as shown in a cohort study from Sweden, the suicide rate among transgender persons is still significantly increased after GAS compared to data from the non-selected general population [34]. This study was also able to show, that the overall mortality of trans-people is increased, so that deaths in the context of diseases are also a possible explanation for non-response.

Nevertheless, it should be noted that of a total of 60 persons, there is no statement about satisfaction with the breast or long-term complications.

Another limitation is the use of the BREAST-Q questionnaire, which is a validated instrument, but validity and reliability were only evaluated in genetic women. Although other studies [9] also used this questionnaire and studies on urological outcome also followed a similar approach, it remains unclear whether validity and reliability are also clearly given in relation to transwomen [19]. However, the use of a specially created questionnaire could not have ensured this any better and with using the BREAST-Q, at least comparability with other studies remains given, even if the results must be interpreted with caution. Further studies should be conducted to verify the results using the GENDER-Q questionnaire as soon as it is available [16].

## Conclusion

The presented study was able to show, that the majority of patients were very satisfied with the results of breast augmentation and that all patients without exception stated that their quality of life had improved as a result of the surgery. In combination with the low complication rates of the operation, a good risk–benefit ratio can be assumed. Furthermore, with regard to the low rate of capsular contracture, new approaches to further understand the etiology of this complication and to further reduce its incidence were shown.
